# Would a comprehensive hearing aid fitting process lead to placebo effects compared to a simple process?

**DOI:** 10.3389/fauot.2024.1411397

**Published:** 2024-06-06

**Authors:** Yu-Hsiang Wu, Megan Dorfler, Elizabeth Stangl, Jacob Oleson

**Affiliations:** 1 Department of Communication Sciences and Disorders, The University of Iowa, Iowa City, IA, United States; 2 Department of Biostatistics, The University of Iowa, Iowa City, IA, United States

**Keywords:** ecological momentary assessment, placebo effect, hearing aid, fitting, outcome

## Abstract

**Objectives:**

Placebo effects refer to the impact of a treatment on health outcomes that cannot be attributed to the treatment itself. The current study aimed to investigate whether a comprehensive hearing aid fitting process would induce placebo effects compared to a simple process, and whether personal attributes such as personality traits could predict susceptibility to these effects.

**Design:**

Thirty adults with hearing loss completed the study. The study began with a fitting session in which the field trial hearing aid configuration (the actual fitting) was set, followed by two experimental conditions. Each condition involved a fake hearing aid fitting and a 3-week field trial. In the fake fitting, bilateral hearing aids were fitted using the Comprehensive protocol (CM) that included multiple assessments and probe-microphone verification or the Streamlined protocol (ST) that did not involve any assessments other than a hearing test. The same hearing aid amplification settings established in the actual fitting, rather than the settings from the fake fittings, were used in the field trials for both conditions. Patient outcomes were measured using the International Outcome Inventory for Hearing Aids (IOI-HA), which was administered as both retrospective self-reports and ecological momentary assessment (EMA) surveys. Personality was assessed using the NEO Five-Factor Inventory. Upon completion of the study, participants expressed their hearing aid preferences based on hearing aids’ real-world performances (prefer CM, prefer ST, or no preference).

**Results:**

For both retrospective self-reports and EMA, the IOI-HA scores of the CM and ST conditions did not significantly differ. Among the 30 participants, 22 expressed a preference for either CM (*n* = 14) or ST (*n* = 8). Younger participants and those with higher levels of agreeableness were more likely to have a hearing aid preference.

**Conclusions:**

At the group level, comprehensive hearing aid fitting process did not generate a placebo effect leading to better outcomes compared to a simple process. However, despite the absence of differences in hearing aid settings, most (73%) participants were affected by placebo effects, believing that one fitting process yielded better real-world outcomes than the other. Personal attributes including personality traits and age are associated with susceptibility to placebo effects.

## 1 Introduction

Placebo effects refer to the change in psychological or physiological symptoms that can be attributed to receiving a substance or undergoing a procedure, even though the inherent power of that substance or procedure may not account for these changes (Geers and Rose, 2011; Kaptchuk and Miller, 2015). The change in health outcomes due to placebos arises from an individual’s perceptions, interpretations and expectations generated by therapeutic activities (Geers and Rose, 2011).

Placebo effects, when employed appropriately, can contribute positively to overall treatment success (Kaptchuk and Miller, 2015). However, they also have the potential to introduce biases in research findings. It has been suggested that placebo effects should be avoided or carefully controlled for in research, particularly in studies aimed at determining the inherent effectiveness of interventions, such as hearing aids (Dawes et al., 2013; Naylor et al., 2015).

Placebo effects have been examined in hearing aid research, with researchers investigating influences that go beyond mere “placebo,” such as the effect of labeling and narratives associated with hearing aids. In this line of research, investigators manipulate participants’ interpretations and expectations about hearing aids, while ensuring that the amplification profile and feature settings of hearing aids remain identical across experimental conditions. Bentler et al. (2003) conducted a field trial to examine the impact of labeling on hearing aid outcomes. Research participants were informed that they would wear either “digital” or “conventional” hearing aids. However, all the hearing aids used were digital (and identical in a subgroup of participants). The results revealed that participants reported better real-world outcomes with the “digital” hearing aids, supporting a placebo effect. The effect sizes were small to medium (Cohen’s *d* = ~0.4). In the laboratory studies by Dawes et al. (2011, 2013), hearing aids were described as either “new” or “conventional,” despite both being programmed identically. The findings indicated that the “new” hearing aids led to higher speech recognition scores, better sound quality ratings, and were preferred by participants. More recently, Rakita et al. (2022) examined the effect of narratives of hearing aids designed to evoke positive, negative, or neutral expectations about the devices on patient outcomes. Despite the hearing aid settings being identical across the three narrative conditions, participants performed better on speech recognition tests and rated the hearing aids more favorably when exposed to positive narratives of hearing aids. Collectively, these studies demonstrate how an individual’s perceptions, interpretations, and expectations about hearing aids can influence hearing aid outcomes.

Notably, Naylor et al. (2015) took a unique approach to examine the impact of narratives embodied in the hearing aid fitting process on patient outcomes. Participants were fitted with hearing aids using two different protocols. In the “interactive” condition, participants actively participated in decision-making with their audiologists. Conversely, in the “diagnostic” condition, patients were passive, with no input or response required from them. Despite differing fitting processes, the hearing aids were always programmed with the NAL-NL1 prescription (Byrne et al., 2001) in the subsequent field trials of both conditions. Hearing aid outcomes were measured after each field trial and participants reported their preference (prefer interactive, prefer diagnostic, or no preference) at the study’s conclusion. The preference question (“Overall, which hearing aid do you prefer?”) was framed to pertain to the hearing aid outcome of the fitting process rather than the process itself. The findings revealed that, across the two experiments, 34 out of 40 participants expressed a clear preference for one of the two fittings, indicating that they were influenced by narratives embodied in hearing aid fitting process and believed that the two processes generated different results. Instead of comparing the two fitting conditions (interactive vs. diagnostic), Naylor et al. (2015) focused on comparing the self-reported outcomes of participants’ preferred vs. non-preferred hearing aids. The findings revealed that the preferred fitting yielded higher ratings of hearing aid benefit and reduced hearing disability compared to the nonpreferred fitting, with medium effect sizes (Cohen’s *d* from 0.3 to 0.68).

### 1.1 Hearing aid fitting process and placebo effects

As previously mentioned, Naylor et al. (2015) used narratives embedded in hearing aid fitting process to manipulate an individual’s perception about hearing aids. Despite differences in patient-audiologist interactions, both fitting protocols (interactive and diagnostic) used in their study took approximately the same amount of time (1 h). Building upon the work of Naylor et al. (2015), the present study aimed to investigate whether a more comprehensive and lengthier hearing aid fitting protocol would produce a placebo effect compared to a simpler and briefer protocol. This investigation was motivated by the work of Kochkin et al. (2010), who analyzed data from MarkeTrak VIII and discovered that an increased number of steps involved in the hearing aid fitting process were associated with higher levels of hearing aid satisfaction. These findings have often been cited as supporting evidence for hearing aid best practice guidelines recommended by professional societies (e.g., American Academy of Audiology, 2006). However, it remains uncertain whether these findings are biased by a potential placebo effect. This uncertainty arises from the observation that patients tend to perceive clinicians who administer a more comprehensive hearing aid fitting protocol, involving more steps, as more knowledgeable, professional, and empathetic (Kochkin et al., 2010). Moreover, increased time spent with clinicians has been shown to correlate with higher patient satisfaction (Lin et al., 2001; Anderson et al., 2007; Patterson et al., 2017). Therefore, the perceptions of increased clinician expertise and professionalism, combined with the longer time spent during a comprehensive fitting process, may induce a placebo effect, leading to hearing aid outcomes surpassing what can be solely attributed to the fitting process itself. Supporting this hypothesis, research has found that the perception of patient-clinician relationships could yield placebo effects. For example, perceptions of the patient-clinician sociocultural (Losin et al., 2017) and racial/ethnic (Anderson et al., 2020) similarity could reduce the pain associated with painful medical procedures.

### 1.2 Personal characteristics and placebo effects

Previous research has shown that personal characteristics such as personality traits, age, and gender could influence an individual’s susceptibility to placebo effects. For instance, individuals with higher levels of agreeableness, characterized by traits such as cooperativeness, empathy, and trust in others, tend to be more susceptible to placebo effects (Jakšić et al., 2013). Similarly, Rakita et al. (2022) discovered that individuals with higher levels of agreeableness were more susceptible to placebo effects, rating hearing aids more favorably when exposed to a positive description about the devices. Conversely, individuals with higher levels of neuroticism, which involves a disposition to experience negative affects like anger, anxiety, and irritability, are less responsive to placebo effects (Jakšić et al., 2013). Regarding age and gender, while some meta-analyses suggest that younger age and female gender are associated with higher susceptibility to placebo effects, the evidence remains mixed across studies (Weimer et al., 2015).

### 1.3 Study objectives

The first objective was to investigate whether a more comprehensive and lengthier hearing aid fitting process, which included multiple assessments and probe-microphone verification, would result in a placebo effect in comparison to a simpler and briefer fitting process. We hypothesized that despite the settings and coupling of hearing aid used in real-world trials being identical across both fitting conditions, the comprehensive fitting would yield superior self-reported outcomes relative to the simple fitting. The second objective was to explore whether personal characteristics including personality traits, age, and gender would be associated with susceptibility to placebo effects evoked by hearing aid fitting process. It was hypothesized that susceptibility to these effects would vary as a function of personality traits, such as agreeableness and neuroticism, age, and gender. We defined that a participant was influenced by placebo effects if this individual believed that the real-world outcome of one fitting was better than the other, despite the absence of differences in hearing aids and the settings during the field trials of both fitting conditions.

## 2 Materials and methods

### 2.1 Overview

**FIGURE 1:**
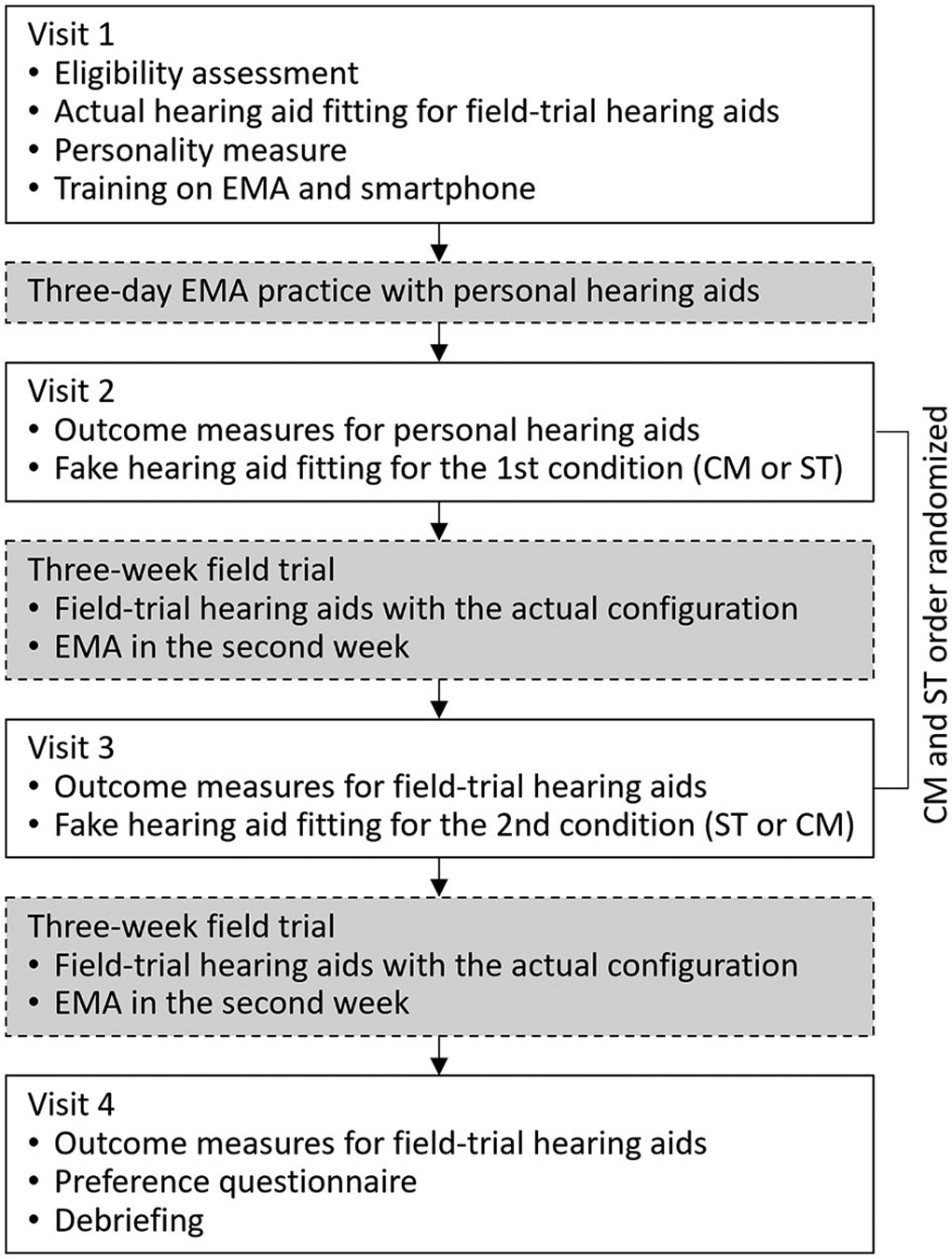
Flow chart of the study. EMA, ecological momentary assessment; CM, Comprehensive fitting; ST, Streamlined fitting.

The study utilized a crossover repeated measures design with deception. Participants were unaware of the true objective of the study (i.e., placebo effects) and were informed that the purpose of the study was to compare two different hearing aid fitting processes. The study started with a laboratory visit in which hearing aid settings for the subsequent field trials were established (i.e., actual fitting). Following this, two experimental conditions were implemented wherein participants’ perceptions about hearing aids were manipulated. In each condition, participants experienced one of the two hearing aid fitting narratives (i.e., fake fitting) and a 3-week field trial. The two fitting narratives were the Comprehensive (CM) fitting that included multiple assessments and probe-microphone verification and the Streamlined (ST) fitting that did not involve any assessments other than a hearing test. Hearing aid settings determined in these fake fittings were not used in the subsequent field trials. Instead, the identical hearing aid setting established in the actual fitting was used in the field trials of both conditions. Participants were led to believe the settings used by the hearing aids were the result of the fake fitting. Additionally, each participant used the same hearing aid pair throughout the study. Patient-reported outcome measures were administered in each experimental condition. At the end of the study, participants completed a questionnaire to indicate their preferred hearing aids. Figure 1 provides an overview of the study flow, while additional details are available below.

### 2.2 Participants

**FIGURE 2:**
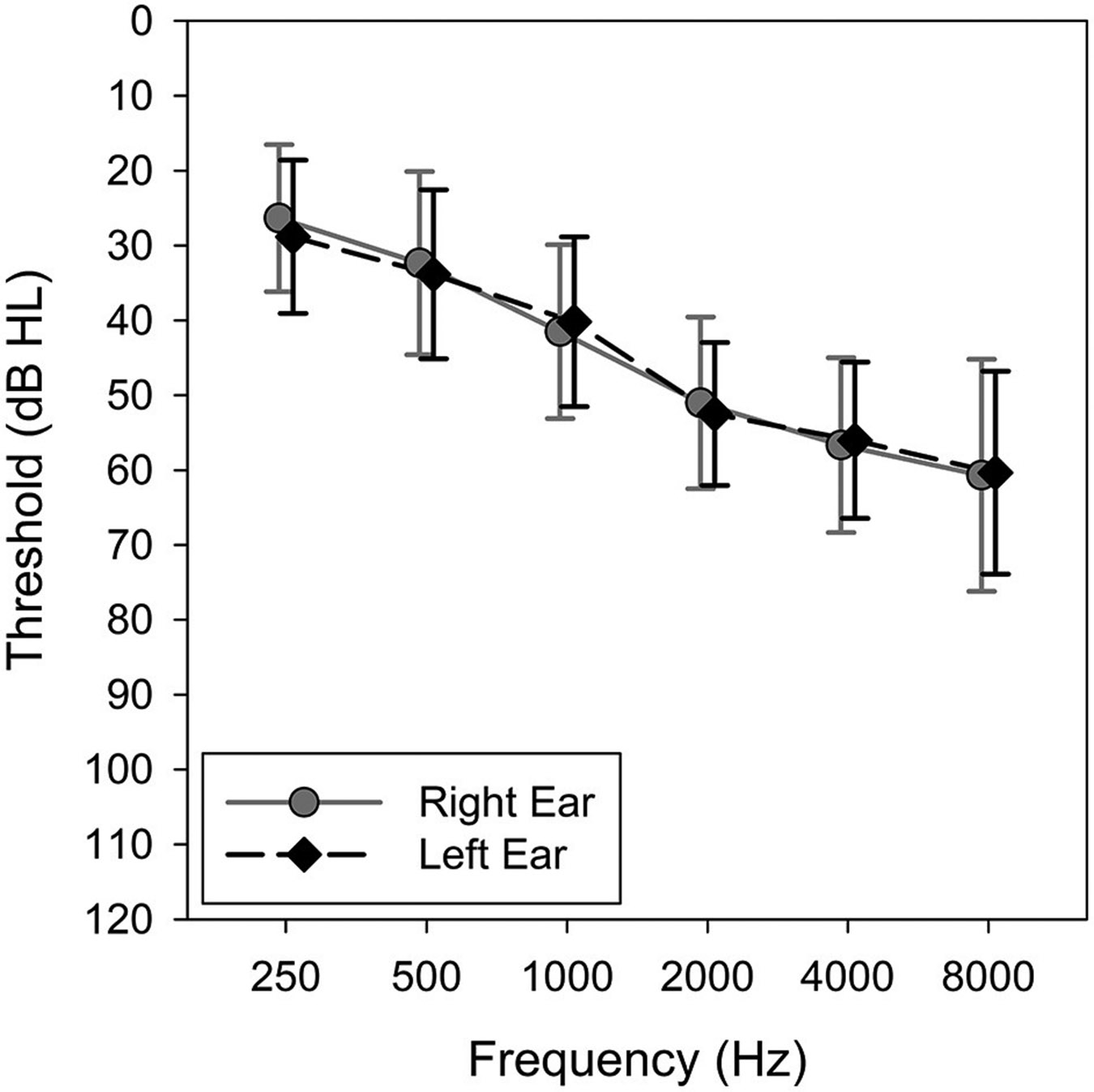
Average audiograms for left and right ears of study participants. Error bars = 1 SD.

The research protocol was approved by the University of Iowa Institutional Review Board, and written informed consent was obtained from all participants. Thirty adults (10 males and 20 females) completed the study. Their ages ranged from 41 to 83 years with a mean of 68.1 years (SD = 9.2). Participants were eligible for inclusion if they met the following criteria: (1) bilateral sensorineural hearing loss defined as pure-tone average at 500, 1,000, and 2,000 Hz ≥ 25 and ≤ 55 dB HL; (2) a minimum of 6-months of consecutive hearing aid experience prior to the study; and (3) ability to understand the study goals, procedures, and perform experiment-related tasks. Participants without prior hearing aid experience were excluded because they tend to exhibit an order effect, showing a preference for the hearing aids they have experienced more recently while experienced hearing aid users do not (Naylor et al., 2015). The mean pure-tone thresholds are shown in Figure 2.

### 2.3 Hearing aids and actual fitting

The study hearing aids used were Hansaton Jam 3-RS13 P behind-the-ear hearing aids. All participants were fitted with the same model of hearing aids. The hearing aids were equipped with four program memories, eight-channel wide dynamic range compression and features including adaptive directional microphones, digital noise reduction algorithms, and impulse sound reduction. The hearing aids also had a datalogging feature that recorded average device daily use (hours per day) and environment classifications (quiet vs. noisy environments).

Prior to conducting the two experimental conditions (CM and ST), actual hearing aid fitting took place. The goal of this fitting was to ensure that participants could comfortably wear the hearing aids during the field trials without encountering any issues. The research audiologist masked the true purpose of the fitting, presenting it as a procedure to determine the suitability of the study hearing aids for their hearing loss. During the fitting session, hearing aid coupling was chosen by the audiologist to minimize feedback issues and ensure a comfortable fit. The first-fit settings were employed, and the gains were set to 100% acclimatization. We opted not to configure the hearing aids with validated prescriptive formulas (e.g., NAL-NL2; Keidser et al., 2011) to prevent the hearing aids from reaching their maximum performance level. This would facilitate a comparison of outcomes between the two experimental conditions. The feedback cancellation feature was enabled, and the audiologist ensured that participants experienced no feedback while wearing the hearing aids. Only one program was activated, which automatically switched between omnidirectional and directional microphones. Other hearing aid features (e.g., noise reduction) were left at the default setting. The volume control was deactivated to ensure consistent amplification throughout the study. No probe microphone measure were used to verify the gains. Once the actual fitting process was completed, participants were informed that the study hearing aids were suitable for their hearing loss. The hearing aid settings established in this fitting session were used in the field trials of both the CM and ST conditions. Additionally, each participant used the same hearing aid pair and coupling in both field trials. These hearing aids were referred to as the *field-trial hearing aids* in the present paper. Throughout the study, no counseling or troubleshooting on the hearing aids was offered.

### 2.4 Comprehensive fitting

This fake fitting consisted of (1) Client Oriented Scale of Improvement questionnaire (COSI; Dillon et al., 1997) to evaluate individual listening needs, (2) Acceptable Noise Level test (ANL; Nabelek et al., 1991) to measure background noise acceptance, (3) measurement of loudness discomfort levels (LDL; Cox, 1995; Ricketts et al., 2017) to determine sound levels that are uncomfortably loud, (4) QuickSIN (Killion et al., 2004) to assess speech recognition performance in noise, (5) probe-microphone real-ear measures to verify the gains of the hearing aids, and (6) hearing aid tuning based on the participant’s feedback.

To provide a clear sense of the duration of the fitting process, the fitting started with the audiologist presenting slides that described the procedure’s characteristics to participants. The CM fitting was described as simulating how hearing aids may be fit by a practicing audiologist who adheres to best practice guidelines. Prior to conducting each measure during the fitting process, participants were provided with information about its purpose and how the results would be used to optimize the hearing aids’ settings. For example, participants were told that the responses from the COSI would be used to configure the automatic adaptive program. The information obtained from the ANL and QuickSIN tests would be utilized to adjust the microphone directionality. LDL would assist in determining the maximum output level of the hearing aids.

Next, the research audiologist programmed a second, decoy set of hearing aids, rather than the field-trial hearing aids, based on the results of the measures described above. Probe microphone measures were also conducted. Whenever feasible, the research audiologist ensured that the hearing aid programming and adjustment procedures were visible to participants. For example, the audiologist deliberately entered participants’ audiograms and LDLs into the fitting software and a probe-microphone hearing aid analyzer (Audioscan Verifit 2; Dorchester, Ontario, Canada) in front of them. The decoy hearing aids were programmed to match real-ear aided response (REAR) targets specified by the NAL-NL2 prescriptive formula (Keidser et al., 2011). The audiologist then fine-tuned the decoy hearing aids based on the participant’s feedback until the participant expressed satisfaction with the sound quality of the hearing aids. The audiologist then informed the participants that the fitting was complete. The CM fitting took ~45 min to 1 h to complete. The decoy hearing aids fitted during this CM fitting, however, were not used in the subsequent field trial. Instead, the field-trial hearing aids described earlier were used in the field trial. See more details in the Procedures section below.

### 2.5 Streamlined fitting

The ST fitting involved a first-fit setting of the hearing aids. Again, the fitting started with the audiologist presenting slides that described the procedure’s characteristics. Participants were informed that this fitting would be based solely on their audiograms without any additional measures taken into consideration and that the goal of the fitting was to simulate a more cost-effective, concise style of programming hearing aids for an individual.

During the ST fitting, participants were seated in front of a computer. The fitting software was opened, and participants’ audiograms were entered. Although the decoy hearing aids were connected to the fitting software, they were not placed on participants’ ears. The research audiologist then selected the first-fit option, causing the visible adjustment of gain on the computer screen. Participants were then informed that the hearing aid fitting had been completed. The ST fitting typically took ~5 min to finish. Like the CM fitting, the decoy hearing aid fitted in the ST fitting were not used in the field trial.

### 2.6 Laboratory tests

#### 2.6.1 Probe microphone measures

To verify if the field-trial hearing aids delivered consistent amplification during the real-world trials of the CM and ST conditions, as-worn REAR was measured post-trial, using a probe microphone and the Verifit 2 hearing aid analyzer with an input speech of 65 dB SPL. The as-worn aided Speech Intelligibility Index (SII; American National Standards Institute, 1997) was also measured using the Verifit 2.

#### 2.6.2 Hearing in noise test

The Hearing in Noise Test (HINT; Nilsson et al., 1994) was used to measure participants’ aided speech recognition performance. Because a previous field trial (Bentler et al., 2003) did not demonstrate a placebo effect using objective tests such as speech recognition tests, we included the HINT to verify the consistency of hearing aid gain-frequency responses between the CM and ST conditions, rather than trying to capture placebo effects. Therefore, we conducted the HINT in quiet rather than in noise, as the HINT in quiet is more sensitive in detecting changes in hearing aid gain-frequency responses (Brody et al., 2018).

The aided HINT was conducted in a sound booth posttrial. The HINT sentences were presented from 0° azimuth to participants without noise. Participants were instructed to repeat a block of 20 HINT sentences. The speech level was adjusted adaptively, depending on the participant’s responses, using the one-up-one-down procedure. The presentation level of the final 17 sentences was averaged to obtain the HINT score.

### 2.7 Outcome measures

#### 2.7.1 International Outcome Inventory for Hearing Aids

The IOI-HA (Cox and Alexander, 2002) consists of seven items that assess seven domains related to the effectiveness of hearing aids: device daily use, benefit, residual activity limitation, satisfaction, residual participation restriction, impact on others, and quality of life. Each item has five responses. Possible scores for each item range from 1 to 5, with higher scores indicating better outcomes. The IOI-HA was the primary outcome of the present study, as it demonstrated the largest effect size in capturing placebo effects compared to other retrospective questionnaires (Naylor et al., 2015, Experiment 1). Because the IOI-HA described here was a retrospective self-report, it is referred to as the Retro-IOI-HA.

The IOI-HA was also administered as an ecological momentary assessment (EMA) survey. EMA, which repeatedly prompts respondents to report their immediate or recent experiences in real-world environments (Shiffman et al., 2008), was included in the present study because it has higher sensitivity than retrospective questionnaires in detecting real-world outcome differences of hearing aids (Wu et al., 2020). We implemented EMA using an application (app), AudioSense (Hasan et al., 2013), on the participant’s own smartphones. The original wording and response options of the IOI-HA were modified to make it suitable for EMA (see Table 1). This adapted version of the IOI-HA was referred to as the EMA-IOI-HA in the current paper. It is important to note that the EMA-IOI-HA questions were presented adaptively. If participants indicated in the first question that they had not used the study hearing aids in the past 3 h, the subsequent six questions would not be presented.

During the 3-week hearing aid trial of each fitting condition, EMA was administered in the 2nd week for 7 days. The EMA app prompted participants to complete surveys at random intervals, approximately every 3 h, within their specified daily time window. If participants missed a survey, they were instructed to wait until the next survey. The EMA results were automatically uploaded by the app to a server located at the University of Iowa.

#### 2.7.2 Hearing handicap inventory for the elderly or for the adult

The HHIE/A (Ventry and Weinstein, 1982; Newman et al., 1990) is a 25-item questionnaire designed to assess the social and emotional impact of hearing loss on an individual’s life. The inventory consists of two subscales: Social subscale (how an individual’s social life is affected by their hearing loss) and Emotional subscale (how hearing loss influences emotional responses). The total score is obtained by adding the scores for all 25 items. Higher scores indicate a greater level of handicap (poorer outcome) caused by the hearing loss. The HHIE was used for participants aged 65 years and over, while the HHIA was administered for participants below that age.

#### 2.7.3 Abbreviated profile of hearing aid benefit

The APHAB (Cox and Alexander, 1995) is a 24-item questionnaire designed to assess the benefit derived from hearing aid use and quantify the level of communication difficulty experienced in various situations due to hearing loss. The questionnaire consists of four subscales: Ease of Communication (speech understanding in favorable listening conditions), Background Noise (speech understanding in settings with high levels of background noise), Reverberation (speech understanding in environments with reverberation), and Aversiveness (an individual’s response to unpleasant environmental sounds). The global score is calculated as the mean of the scores for the Ease of Communication, Background Noise, and Reverberation subscales. Higher scores indicate a greater degree of communication difficulty (poorer outcome).

### 2.8 Preference questionnaire

At the conclusion of the study, participants completed the Preference Questionnaire adapted from Naylor et al. (2015) to indicate their hearing aid preference by selecting one of three options: the first fitting, the second fitting, or no preference. They were instructed to base their preference solely on the hearing aid performances themselves, disregarding the fitting process. Participants also rated the certainty of their preference on a scale from 1 to 10, with 10 representing the highest level of certainty. Lastly, participants were asked to provide reasons for their preference.

### 2.9 Personality measure

Personality was assessed using the NEO Five-Factor Inventory (NEO-FFI; McCrae and John, 1992). This inventory consists of 60 items and evaluates five subscales representing fundamental dimensions of normal personality: openness (curiosity, interest, and insightfulness), conscientiousness (efficiency, reliability, and thoroughness), extraversion (enthusiasm, talkativeness, and action), agreeableness (appreciation, trust, and compliance), and neuroticism (anger, anxiety, and impulsivity). Scoring on the NEO-FFI involves transforming raw scores for each trait into standardized scores, with gender-specific norms available.

### 2.10 Procedures

The study spanned 7 weeks and involved four laboratory visits (Figure 1). During the first visit, participants were informed that the study aimed to compare two different hearing aid fitting processes (CM and ST). After obtaining consent, participants’ hearing thresholds were measured using pure-tone audiometry. If participants met the inclusion criteria, the actual hearing aid fitting that created the settings for subsequent field trials took place. The settings were saved in the field-trial hearing aids which were then placed in the participants’ research folders. The hearing aids would later be used in both field trials. After the fitting, the participants were briefly oriented to the study hearing aids. Next, the NEO-FFI was administered. The EMA app was installed on participants’ smartphones and training on the app was provided. Once participants demonstrated competence in EMA tasks, they returned home and began a 3-day EMA practice session. During the practice session, participants were instructed to wear their personal hearing aids and complete EMA surveys.

Participants returned to the laboratory for the second visit after the practice session. If any participants had difficulty with EMA or smartphone-related tasks during the practice session, they received additional instructions to ensure proper usage. Next, to ensure that both the second and third visits had a comparable duration of conducting outcome measures, laboratory tests (probe microphone measures and the HINT) and questionnaires (Retro-IOI-HA, HHIE/A, and APHAB) were administered to assess the performance of participants’ *personal* hearing aids. If the second visit did not include these measures, it could be significantly shorter than the third visit (see Figure 1), potentially impacting how participants perceive the fake fitting processes included in these two visits. The outcome data of participants’ personal hearing aids were not included in the analysis. Following the completion of measures on participants’ personal hearing aids, one of the two fitting conditions (CM or ST) commenced, with the order randomized among participants. The decoy hearing aids were fitted using the procedures described above. Upon completion of the fitting, participants were instructed to take a break, during which time they were informed that the audiologist would be preparing the hearing aids for home use. Unaware to participants, during this interval the audiologist switched out the decoy hearing aids with the field-trial hearing aids. Participants received the field-trial hearing aids and were instructed to wear the hearing aids for the next 3 weeks. EMA surveys were scheduled to begin during the 2nd week and continued for 7 days. The week between the end of EMA and the next visit served as a washout period, minimizing the likelihood that the experience of EMA-IOI-HA influenced the subsequent Retro-IOI-HA assessment.

After completing the 3-week field trial, participants returned to the laboratory for the third visit. Hearing aid datalogging data were retrieved from the devices. Laboratory tests (as-worn REAR and aided HINT) and retrospective questionnaires were administered to measure the outcomes of the field-trial hearing aids. Next, the second condition was initiated and the fake fitting was performed on the decoy hearing aids. Before participants left the laboratory, the decoy hearing aids were replaced by the field-trial hearing aids. The second 3-week field trial, involving EMA surveys conducted throughout the 2nd week, was then started.

Participants returned to the lab for the fourth visit after the 3-week field trial. Once again, laboratory tests and retrospective questionnaires were administered. Upon completion, participants filled out the Preference Questionnaire. Participants were then debriefed regarding the true objectives of the study. Participants returned the field-trial hearing aids and were compensated for their time.

## 3 Results

### 3.1 Consistency in hearing aid setting, usage, and listening environments

**FIGURE 3:**
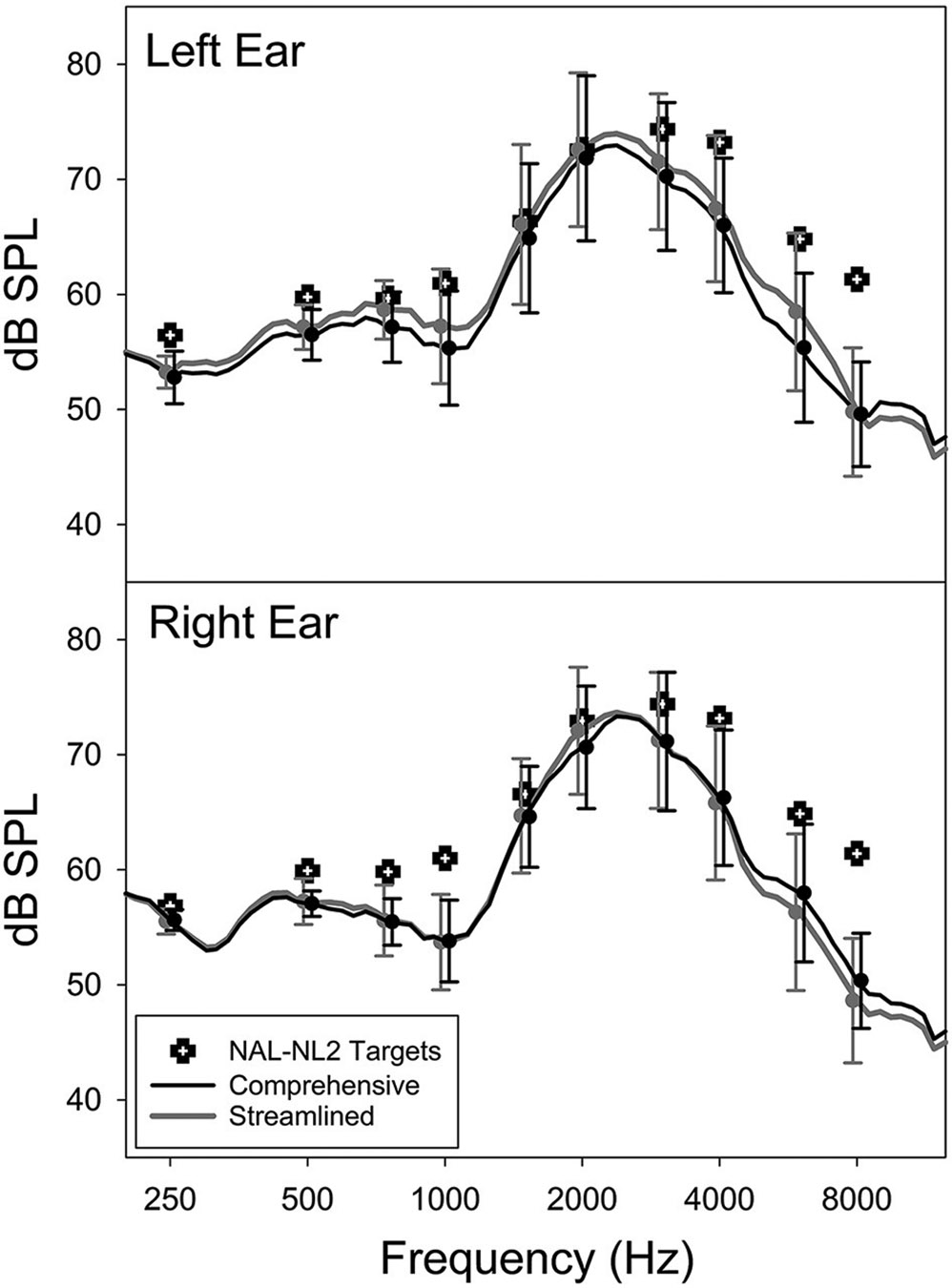
Average as-worn real-ear aided response (REAR) and NAL-NL2 targets of the Comprehensive and Streamlined fitting conditions.

Figure 3 shows the mean post-trial as-worn REAR of the CM and ST conditions averaged across all participants, measured using a 65-dB SPL speech input. The figure also includes the mean REAR targets prescribed by NAL-NL2. Overall, the field-trial hearing aids under-amplified sounds compared to the NAL-NL2 targets, especially at frequencies above 2,000 Hz. Notably, at the group level, the mean REAR of the CM fitting highly resembled that of the ST fitting. The standard deviation of the absolute REAR difference between the CM and ST conditions at 250, 500, 1,000, 2,000, 4,000, and 6,000 Hz was 1.3, 1.5, 2.3, 2.2, 2.5, and 4.5 dB, respectively. These data were in a good agreement with previous reports of REAR test-retest reliability (Hawkins and Mueller, 1992), suggesting that, at the individual level, the REARs for both CM and ST fittings were similar. The mean aided SIIs of the CM and ST conditions were 52.0% (SD = 12.6%) and 53.4% (SD = 13.5%) for left ear and 49.7% (SD = 14.5%) and 49.0% (13.8%) for right ear, respectively. The difference between the two fittings was not statistically significant (paired *t*-test, left ear: *t* = −1.9, *p* = 0.073; right ear: *t* = 0.9, *p* = 0.379). The absolute SII difference between CM and ST ranged from 0 to 10% with a mean and median of 3.0 and 2.0%, respectively. Collectively, these results indicated that the study hearing aids delivered consistent amplification in the field trials of both conditions.

The consistency of hearing aid settings was also examined using the HINT—a behavioral measure. The aided HINT scores of the CM and ST conditions were 38.2 dBA (SD = 7.9) and 37.5 dBA (SD = 7.65), respectively. The difference is not statistically significant (paired *t*-test, *t* = 1.44, *p* = 0.159). The absolute difference in the HINT score between CM and ST ranged from 0 to 8.6 dB, with a mean and median of 2.0 and 1.1 dB, respectively. At the individual level, the HINT score for CM and ST fittings did not significantly differ in 22 out of 30 participants, as determined by the 95% critical difference norm of the HINT in quiet (1.94 dB; Nilsson et al., 1994). Because the inter-session variation for people with hearing loss is expected to be larger than the intra-session critical difference norm established using people with normal hearing, we believe that the HINT scores between the CM and ST conditions did not show significant differences for the majority of our participants.

Datalogging data were available from 24 participants after removing unreasonable data (e.g., average daily use = 22 h) and instances where the data logging was unavailable in the fitting software. The average daily device use time was 10.0 h (SD = 3.1) for the CM condition and 10.1 h (SD = 3.1) for the ST condition, and the difference was not statistically significant (paired *t*-test, *t* = −0.48, *p* = 0.633). The environment classification data indicated that, on average, participants spent 59.3% (SD = 10.5%) and 61.6% (SD = 13.1%) of the time in quiet environments in the CM and ST conditions, respectively. The difference between the two conditions was not statistically significant (paired *t*-test, *t* = 0.085, *p* = 0.933). These results indicated that participants consistently used the fieldtrial hearing aids and encountered similar listening environments in both conditions.

### 3.2 CM vs. ST: primary outcome

To test the hypothesis that the CM fitting would yield placebo effects compared to the ST fitting, we compared the outcome measure results between the CM and ST conditions. We started with the primary outcome of the present study: the IOI-HA. We analyzed both Retro-IOI-HA and EMA-IOI-HA within the same statistical model so that we could not only compare the outcomes of the CM and ST conditions, but also determine whether retrospective self-reports and EMA would reveal similar placebo effects.

In total, 1,235 EMA-IOI-HA surveys were completed across the two conditions (CM: *n* = 614; ST: *n* = 621). Due to technical issues, two participants were unable to complete EMA surveys in one of the conditions. On average each participant completed 3.0 and 3.1 surveys per day in the CM and ST conditions, respectively. The survey response rate was 59.8% (SD = 25.1%) and 60.7% (SD = 27.7%) for the CM and ST conditions, respectively. Recall that the EMA app presented questions adaptively, such that items 2–7 of the EMA-IOI-HA were not displayed when the response to the first question “During the previous 3 h, how much did you use the study hearing aids?” was “Never/Not at all” (refer to Table 1). Therefore, using the average of items 1–7 as the global score would be inappropriate for the EMA-IOI-HA. The “Never/Not at all” option was chosen in 8.1 and 8.2% of the EMA surveys in the CM and ST conditions, respectively. Consequently, in this analysis we excluded item 1 and computed the average scores across items 2–7 as the EMA-IOI-HA global score. The same scoring method was applied to the Retro-IOI-HA. It is worth noting that the EMA involved repeated sampling, resulting in more data points for the EMA-IOI-HA (an average of 21.3 surveys per participant per condition) compared to the Retro-IOI-HA (one assessment per participant per condition). To ensure direct comparison and analyze them within the same statistical model, we averaged the EMA-IOI-HA scores for individual surveys completed by a participant in a particular fitting condition for data analysis.

**FIGURE 4:**
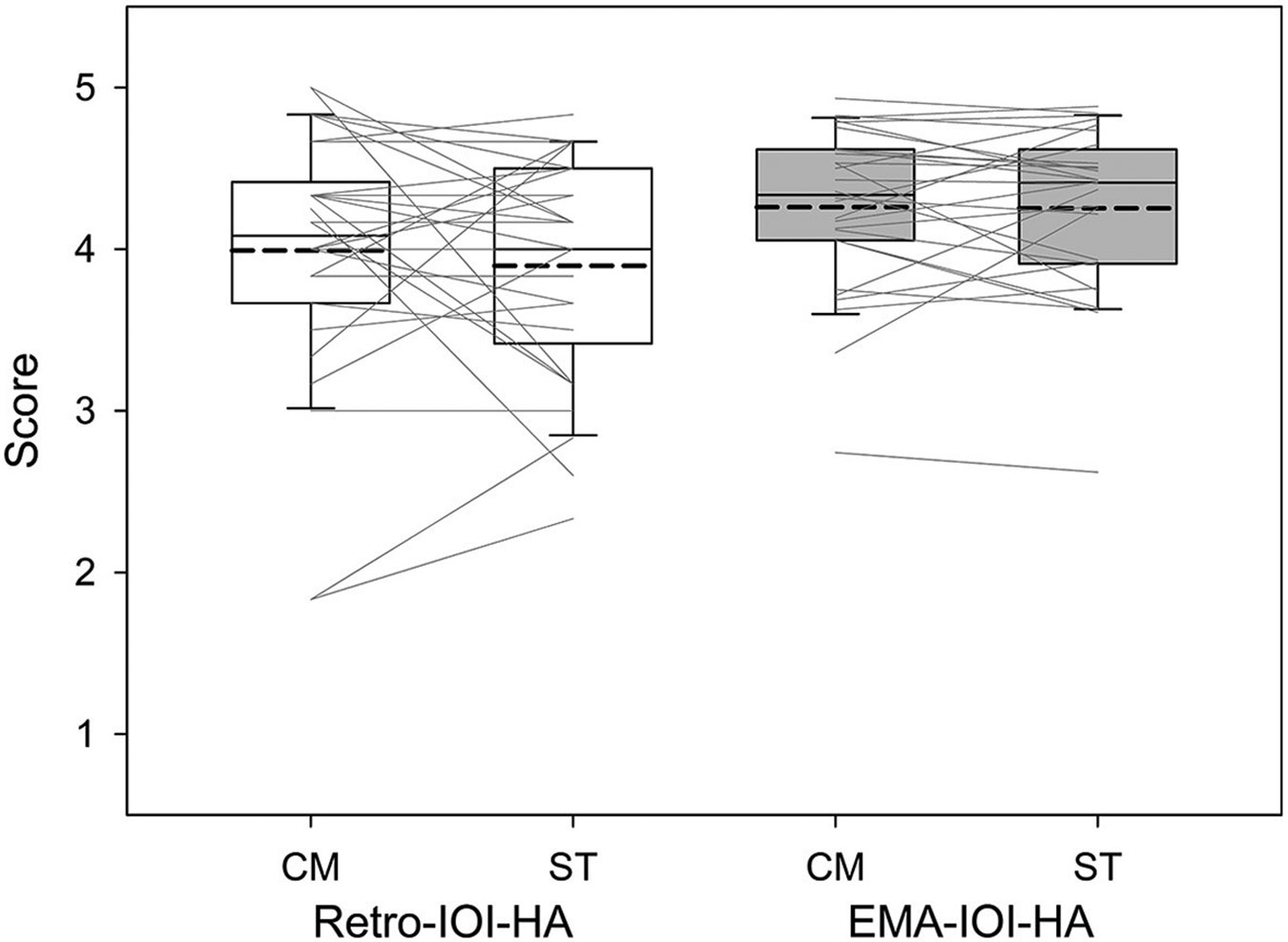
Boxplots of IOI-HA scores based on the Comprehensive (CM) and Streamlined (ST) conditions. Boundaries of the boxes represent the 25th and 75th percentile and error bars indicate the 10th and 90th percentiles. Thick dashed lines represent means.

Figure 4 presents the boxplots for the CM and ST conditions in relation to each measure. To compare outcomes of the CM and ST fittings, we used a regression model with unstructured correlation matrix to account for the correlation due to repeated measures. Fixed effects were fitting (CM, ST), measure (Retro, EMA), and an interaction between fitting and measure. The unstructured correlation matrix allows for all within subjects effects (i.e., CM/Retro, CM/EMA, ST/Retro, and ST/EMA) to have different correlations with each other. The results indicated that Retro-IOI-HA scores were significantly lower than EMA-IOI-HA scores (*t* = −3.14, *p* =0.0021). The IOI-HA score between CM and ST, however, did not significantly differ (*t* = −0.26, *p* = 0.793). The interaction was not significant either (*t* = −0.66, *p* = 0.508).

### 3.3 CM vs. ST: secondary outcomes

**FIGURE 5:**
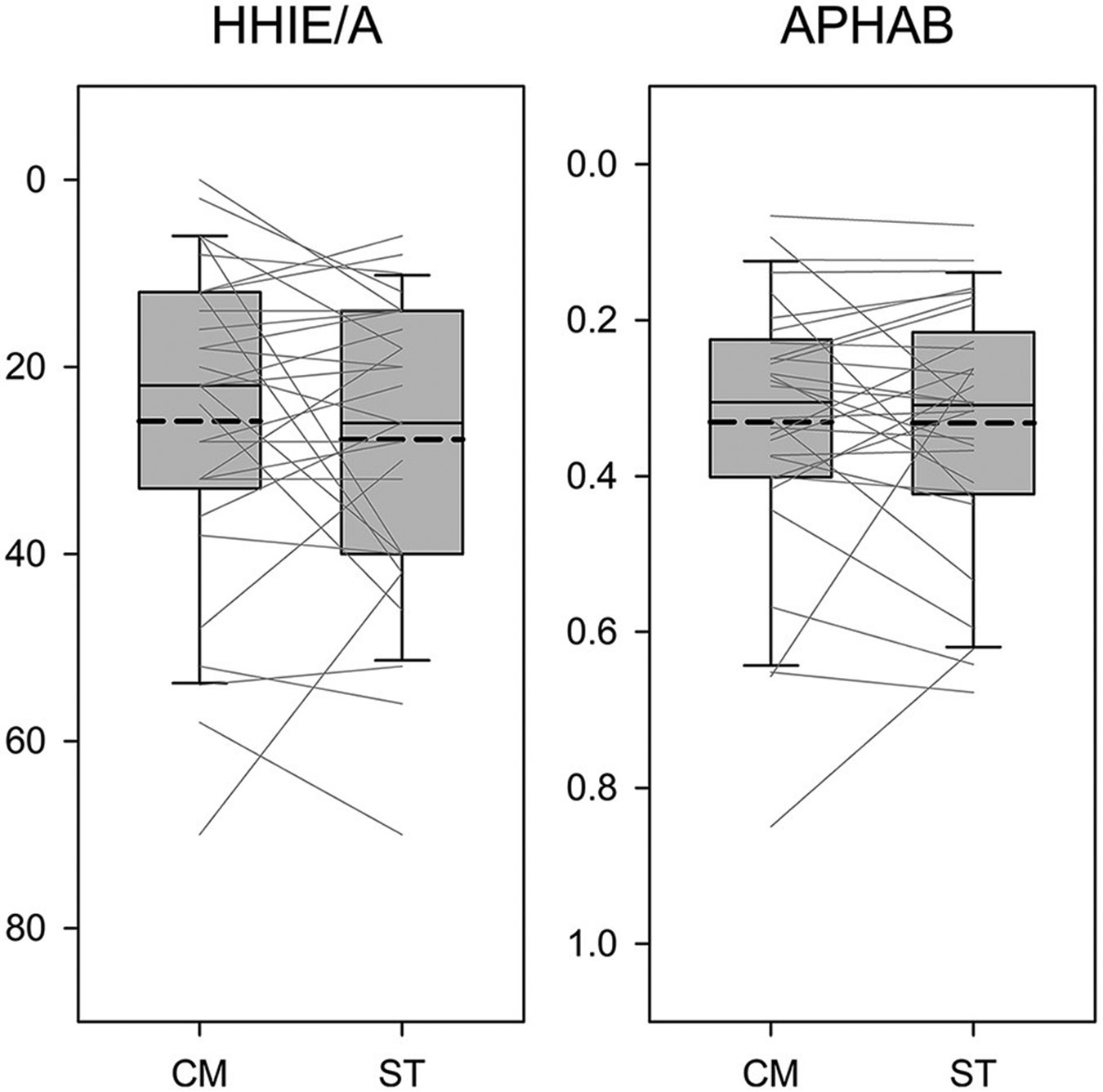
Boxplots of outcome scores as a function of the Comprehensive (CM) and Streamlined (ST) fitting conditions. The y-axis is reversed so that the top of the figure represents better outcomes. Boundaries of the boxes represent the 25th and 75th percentile and error bars indicate the 10th and 90th percentiles. Thick dashed lines represent means.

We then compared the results of the HHIE/A and APHAB between the CM and ST fittings. Figure 5 presents the boxplots for the CM and ST conditions in relation to each measure. The y-axis of the figures has been reversed so that the top of the figure represents better outcomes. Paired *t*-tests indicated the scores of both questionnaires were not significantly different between the CM and ST conditions (HHIE/A: *t* = −0.81, *p* = 0.426; APHAB: *t* = −0.06, *p* = 0.951).

### 3.4 Hearing aid preference

Recall that the Preference Questionnaire specifically inquired about participants’ preference regarding hearing aid performances in the real world without considering the fitting process. Out of the 30 participants, 22 expressed a preference. The certainty of preference, rated on a scale of 1–10, was moderately high (prefer CM: mean = 6.79, SD = 2.69; prefer ST: mean = 7.44, SD = 2.90; no preference: mean = 7.06, SD = 2.98). The reasons provided for the preference were primarily related to sound quality. Examples included statements such as “The first fitting sounded clearer” and “The first hearing aid settings had more success in managing environmental noises.”

Among the 22 participants who had a preference, 12 and 10 participants preferred the first and second fittings, respectively. An exact binomial test indicated that 12 vs. 10 (55% preferred the first fitting) was not significantly different from 50% (*p* = 0.832, no preference excluded). This suggests a lack of evidence supporting an order effect. Moreover, for those who had a preference, 14 preferred CM while eight preferred ST. An exact binomial test indicated that 14 vs. 8 (64% preferred CM) was not significantly different from 50% between preferring CM and preferring ST (*p* = 0.286).

### 3.5 Factors predicting placebo effect susceptibility

Finally, we examined whether personal attributes could predict susceptibility to placebo effects. Susceptibility was quantified by the likelihood of preferring one of the two fittings relative to having no preference, such that higher likelihood indicated greater susceptibility. Predictors included in the analysis were NEO-FFI scores (openness, conscientiousness, extraversion, agreeableness, and neuroticism), as well as age and gender. We combined participants who preferred CM and ST and performed a logistic regression to predict whether an individual had a hearing aid preference or not. Given the relatively large number of predictors compared to the sample size and the intercorrelations among predictors, we performed variable selection by examining all combinations of variables. We selected the model with the smallest Bayesian Information Criterion to identify the best combination of predictors to include in the final model. The final model (Table 2) revealed that individuals with higher levels of agreeableness were more likely to have a preference (*p* = 0.0246), while older participants were less likely to have a preference (*p* = 0.0470).

## 4 Discussion

The purpose of the study was to determine whether a comprehensive hearing aid fitting process would result in placebo effects in comparison to a simple process. The study also explored whether personal attributes could predict susceptibility to placebo effects quantified by the likelihood of preferring one of the two fittings.

### 4.1 Placebo effects

Despite the variations in the number of measures and time duration between the CM and ST fittings, there was no significant difference in the hearing aid outcomes of these two fittings, contrary to our hypothesis. These findings suggest that, at least within the research context, the time and effort dedicated by audiologists to hearing aid fitting processes is unlikely to introduce placebo bias into hearing aid outcomes. Two potential reasons might explain why placebo effects were not observed. First, both CM and ST fittings were conducted by the same audiologist. Therefore, the CM fitting may not have conveyed a perception of greater clinician expertise and professionalism compared to the ST fitting, potentially diminishing the occurrence of placebo effects. Second, even though we deliberately signaled the start and end times of the fitting sessions, participants might not have perceived the time difference between the two fittings due to the considerable amount of time spent conducting outcome measures in each laboratory visit (see Figure 1).

### 4.2 Personal attributes and susceptibility to placebo effects

Although at the group level the outcomes of the CM and ST fittings did not significantly differ, 73% of participants believed that the two fittings yield different real-world outcomes and expressed a preference. This finding is consistent with the study by Naylor et al. (2015), which revealed that hearing aid users could be influenced by narratives embodied in hearing aid fitting process.

Table 2 further demonstrates that personality traits and age are linked to the likelihood of having a preference. Consistent with the literature (Jakšić et al., 2013; Rakita et al., 2022), participants in the current study with higher levels of agreeableness were more likely to be influenced by placebo effects and to have a preference. Also, older participants were less likely to have a preference, indicating that younger adults were more susceptible to placebo effects. This finding is somewhat consistent with existing literature. Specifically, Weimer et al. (2015) evaluated systematic reviews and meta-analyses and found that while only 15 out of the 75 analyses examined reported a positive association between younger age and a higher placebo response, even fewer analyses (*n* = 5) found the opposite.

### 4.3 Retrospective self-reports vs. EMA

Although it is not the main goal of the present study, we found that the EMA-IOI-HA scores were significantly higher (indicating better outcomes) compared to the Retro-IOI-HA scores (Figure 4). This finding is consistent with a study conducted by Wu et al. (2020), which demonstrated that participants reported better hearing aid outcomes when using the EMA version of the Glasgow Hearing Aid Benefit Profile (Gatehouse, 1999) in comparison to the retrospective version. The reason for this discrepancy is not fully understood. One potential explanation could be attributed to a negativity bias, which is a tendency to utilize negative information more than positive information in an individual’s judgment (Vaish et al., 2008). Because this bias is more pronounced when respondents complete retrospective questionnaires than when they complete EMA (Neubauer et al., 2020), the former tend to exhibit poorer patient outcomes.

### 4.4 Clinical implications and study limitations

Our participants did not report better hearing aid outcomes with the CM fitting compared to the ST fitting. The absence of evidence supporting this placebo effect suggests that the association between the number of steps involved in the hearing aid fitting process and hearing aid satisfaction (Kochkin et al., 2010) is likely due to the inherent effectiveness of the procedures, rather than the placebo effects associated with the perceptions of clinician’s professionalism or the duration of procedures. However, our findings should be interpreted cautiously as the fitting was conducted in a research setting. The results may not generalize to a real-world clinical setting wherein patients are required to pay for the services. Also note that the hearing aids used in the field trials were programmed with the first-fit settings and therefore under-amplified sounds compared to the targets prescribed by NAL-NL2 (Figure 3). This under-amplification might have negatively impacted participants’ satisfaction with their hearing aids, preventing them from perceiving clinician’s expertise in the CM fitting. As a result, the occurrence of placebo effects might have been diminished. A more noticeable placebo effect might have emerged if the gain had been set at the prescriptive target instead of the first-fit in both conditions.

Although placebo effects were not found in the current study, literature on placebo effects (Bentler et al., 2003; Dawes et al., 2011, 2013; Naylor et al., 2015; Rakita et al., 2022) indicate that when administering a hearing aid intervention, the overall patient outcomes comprises both the actual effects of the hearing aids and placebo effects. When appropriately implemented, clinicians can utilize placebo effects to enhance patient outcomes. However, it is equally essential for clinicians to recognize that placebos may also lead to negative consequences. This negative impact is known as the *nocebo* effect, representing the adverse impact of a treatment that cannot be attributed to the treatment itself (Kaptchuk and Miller, 2015).

## 5 Conclusions

The present study provided no evidence supporting the hypothesis that a comprehensive hearing aid fitting process, which included multiple tests and probe-microphone verification, would generate a placebo effect leading to more positive outcomes compared to a simple process. However, despite the absence of differences in hearing aids and the settings, over 70% of participants believed that the two fitting processes yielded different real-world outcomes. Finally, participants with younger age and higher levels of agreeableness were more likely to be influenced by placebo effects and preferred one of the two fitting processes.

## Figures and Tables

**TABLE 1:** EMA survey questions and response options.

Questions	Response options
[Item 1] During the previous 3 h, how much did you use the study hearing aids?	Never/Not at all
	About ¼ of the time
	About ½ of the time
	About ¾ of the time
	All the time
[Item 2] Considering the listening situations over the past 3 h, how much have the study hearing aids helped in those situations?	Helped not at all
	Helped slightly
	Helped moderately
	Helped quite a lot
	Helped very much
[Item 3] Consider again the listening situations over the past 3 h. With the study hearing aids, how much difficulty did you STILL have in those situations?	Very much difficulty
	Quite a lot of difficulty
	Moderate difficulty
	Slight difficulty
	No difficulty
[Item 4] Considering the past 3 h, do you think the study hearing aids were worth the trouble?	Not at all worth it
	Slightly worth it
	Moderately worth it
	Quite a lot worth it
	Very much worth it
[Item 5] Over the past 3 h, with the study hearing aids, how much have your hearing difficulties affected the things you can do?	Affected very much
	Affected quite a lot
	Affected moderately
	Affected slightly
	Affected not at all
[Item 6] Over the past 3 h, with the study hearing aids how much do you think other people were bothered by your hearing difficulties?	Bothered very much
	Bothered quite a lot
	Bothered moderately
	Bothered slightly
	Bothered not at all
[Item 7] Considering the past 3 h, how much have the study hearing aids changed your enjoyment of life?	Worse
	No change
	Slight better
	Quite a lot better
	Very much better

**TABLE 2:** Summary statistics of logistic regression models predicting the probability of having a hearing aid preference.

Parameter	Estimate	Standard error	Wald Chi-Square	*P*-value	Odds ratio
Intercept	4.02	7.99	0.25	0.615	
Agreeableness	0.17	0.08	5.05	0.0246	1.19
Extraversion	0.17	0.09	3.61	0.0574	1.18
Age	−0.28	0.14	3.94	0.0470	0.75

## Data Availability

The raw data supporting the conclusions of this article will be made available by the authors, without undue reservation.
